# Epigenetics of Ancient DNA

**Published:** 2016

**Authors:** S. V. Zhenilo, A.S. Sokolov, E. B. Prokhortchouk

**Affiliations:** Institute of Bioengineering, Federal Research Center “Fundamentals of Biotechnology”, Russian Academy of Sciences, prospect 60-letiya Oktyabrya, Str. 7/1, Moscow, 117312, Russia

**Keywords:** epigenetics, ancient DNA, DNA methylation

## Abstract

Initially, the study of DNA isolated from ancient specimens had been based on the analysis of the primary nucleotide sequence. This approach has allowed researchers to study the evolutionary changes that occur in different populations and determine the influence of the environment on genetic selection. However, the improvement of methodological approaches to genome-wide analysis has opened up new possibilities in the search for the epigenetic mechanisms involved in the regulation of gene expression. It was discovered recently that the methylation status of the regulatory elements of the *HOXD *cluster and *MEIS*1 gene changed during human evolution. Epigenetic changes in these genes played a key role in the evolution of the limbs of modern humans. Recent works have demonstrated that it is possible to determine the transcriptional activity of genes in ancient DNA samples by combining information on DNA methylation and the DNAaseI hypersensitive sequences located at the transcription start sites of genes. In the nearest future, if a preserved fossils brain is found, it will be possible to identify the evolutionary changes in the higher nervous system associated with epigenetic differences.

## INTRODUCTION

The study of DNA isolated from archaeological and paleontological specimens provides information about our evolutionary past. Initially, the investigation of ancient DNA had consisted in an analysis of nucleotide sequences. At that stage, researchers encountered a host of difficulties related to the quality of the ancient DNA, its contamination with foreign DNA and others, and these problems were resolved, in particular, by means of improved whole genome sequencing methods. Then, the development of modern sequencing technology has also allowed researchers to analyze the information contained in the epigenetic code. On the one hand, physical characteristics such as susceptibility to a disease, and even some of the psychological characteristics of an individual are determined by genetic factors. On the other hand, it would be a mistake to dismiss the impact of the environment. Gene expression is determined by not only the nucleotide sequence, but also by a number of adaptively regulated processes that lead to changes in the DNA methylation level, histone code, and the spectrum of miRNA. These epigenetic mechanisms are involved in the formation of the chromatin structures required for the regulation of gene expression. With the combination of high-technology sequencing and different methodological approaches, whole genome maps of DNA methylation have been derived in various types of human cells and mice: somatic, stem, germ, cancer, and other types of cells [1].

The central characteristic of any ancient DNA is its degradation and cytosine deamination. Until recently, it was considered impossible to extract information on the transcriptional activity of genes from DNA that had been isolated long after the death of an individual. Nevertheless, in 2010 S. Paabo *et al*. made the first attempts to build methylation maps of ancient DNA, and they demonstrated the potential to determine the *in vivo *patterns of CpG methylation in Neanderthal DNA [2]. Shortly after, by bisulfite allelic sequencing of loci from late Pleistocene bison DNA remains, B. Llamas *et al*. [3] showed that DNA methylation patterns are preserved in ancient DNA.

## METHODS USED TO STUDY ANCIENT DNA METHYLATION

**Fig. 1:**
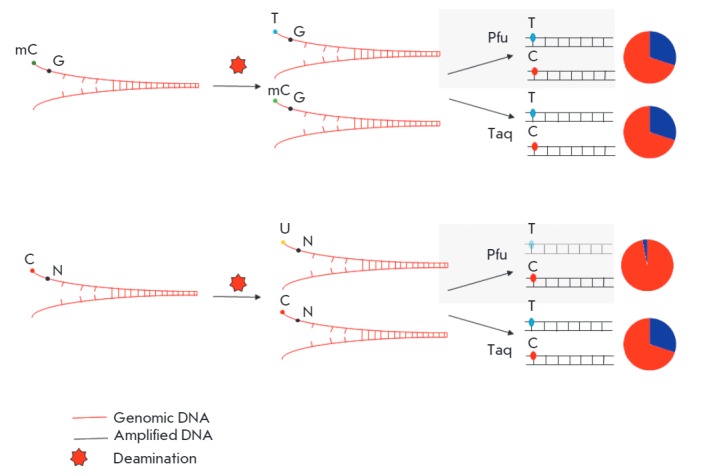
Scheme of cytosine methylation detection in ancient DNA via substitution rates of C-T relative to the original chain during deamination. Taq polymerase is not sensitive to the presence of uracil, and Pfu polymerase cannot continue the chain synthesis in the presence of uracil. Deamination of methylated cytosines (mC) does not affect the ratio of C-T substitutions detected using Taq- and Pfu-polymerases. Deamination of unmethylated cytosines creates “impassable” uracil for Pfu polymerase. Thus, all the readings starting from T (in the context of genomic CG) in NGS libraries prepared using Pfu polymerase definitely indicate the methylation status of cytosine in ancient DNA.

Deamination of methylated cytosine residues and their transformation into thymine, which occurs after death, makes it difficult to perform a quantitative analysis of methylated cytosines. Only as recently as 2014 was a method developed that allowed researchers to conduct a genome-wide methylation analysis of ancient DNA [4-6]. During bisulfite sequencing, unmethylated cytosine residues are chemically converted to uracil residues, which are then read by polymerases, such as Taq-polymerase, in the manner of thymines in PCR. In vertebrate cells, mapping of these C-T-mutations is performed at one nucleotide precision. A similar chemical transformation occurs naturally after death, mainly due to the hydrolytic deamination of the cytosines located in single-stranded areas [7]. The use of Taq-polymerase, for example Taq platinum high fidelity (Hifi), which is insensitive to the presence of uracil, leads to an increase in C-T-substitutions relative to the original strand. These substitutions cannot be observed when Pfu-polymerase is used, such as Phusion. Pfu-polymerase is unable to continue the synthesis of a strand in the presence of uracil. 5-methylcytosine is deaminated to thymine, in contrast to unmodified cytosine, and can be successfully amplified by Pfu-polymerase (*Fig. 1*). Thus, an increase in the number of C-T-substitutions in the analysis of ancient DNA allows one to distinguish methylated cytosines from unmethylated cytosines [5]. Deamination is a stochastic process: so, there is always methylated CpG-dinucleotides (MpG) not deaminated with time that are amplifiable as common CpG (*Fig. 1*). However, this method allows one to determine the methylation of not all cytosines, but only the deaminated ones. To identify methylated cytosines with single nucleotide resolution, it is necessary to increase the coverage depth. R. Smith *et al*. [8] demonstrated the possibility of assessing the single CpG-dinucleotide methylation status located in mobile genetic elements LINE-1 in ancient DNA from the skeletal remains of five North Americans ranging in age from approximately 200 to 4,000 years BC. When there is no need to determine MpG at single nucleotide precision, it is sufficient to analyze the number of CpG-TpG-substitutions at the DNA regions of interest. This approach was applied in reconstructing the DNA methylation maps of the Neanderthal, the Denisovan, and the Paleo-Eskimo human genomes [4]. It was demonstrated that DNA methylation exhibits high conservation over time in bone marrow and in the hair follicle of present-day and ancient humans.

## ANALYSIS OF ANCIENT DNA EPIGENOME

In their study of differentially methylated regions, Gokhman D. *et al*. found that the promoters of genes and the sequences of the *HOXD9 *and *HOXD10 *genes, key regulators of limb development, are methylated in the bone marrow of ancient samples (Neanderthal and the Denisovan humans) and unmethylated in the DNA of present-day humans [4]. In mouse model systems, it was shown that changes in the *HOXD *gene cluster expression, especially the *HOXD9 *and *HOXD10 *genes, lead to morphological changes [9] which resemble the differences in the limb organization of Neanderthals and modern humans. This fact suggests that epigenetic changes in the *HOXD *gene cluster play a key role in the limb evolution of modern humans. Moreover, a differentially methylated region was found within the *MEIS1 *gene encoding a protein that regulates the *HOXD *gene cluster activity [4]. Information on the ancient DNA methylation of large genomic regions via C-T-substitution rates can be used to search for stretches with changes the in methylation level in the bone marrow and hair follicles of our ancestors. Such an analysis will allow us to detect both hypermethylated CpG-islands and also:

1) long (several hundred kbp to several million bp) partially methylated domains (PMD) which do not contain genes and colocalize with lamina-associated domains [10, 11];

2) DNA methylation valleys (DMV) (several kbp), hypomethylated in most tissues containing a large number of developmental genes and regions for transcription factor binding [12, 13], but hypermethylated in colon cancer cells [12];

3) extended regions of low methylation (canyons) (tens of kbp) recently discovered in hematopoietic stem cells [14]; and

4) epigenetic programs of intestinal inflammation that are characterized by hypermethylation of DMV, low CpG density, and active chromatin marks [15].

All of these studies are based on an analysis of the methylation level of genome regions, the length of which varies from several kbp to several million base pairs. Epigenetic analysis of ancient DNA, based on a search for C-T-substitution-rich regions, raises the possibility of assessing the adaptation signals and/or markers of diseases. However, this requires well-preserved tissues (brain, intestine, muscle, blood), which is common for remains found in permafrost, such as the remains of mammoths that lived in the Pleistocene (see below). In 2014, a unique effort was published on an epigenetic map generated from the DNA of one hair follicle that belonged to a Paleo-Eskimo human. It allowed researchers to estimate the age at death of the individual [5]. This was done during a forensic investigation that showed the possibility of determining age based on the methylation level of certain CpG-dinucleotides [16]. Assuming that 6,000 years ago the external environment affected methylation the same way it does today, then based on modern databases one can determine the age of an ancient human. L. Orlando *et al*. showed that the Saqqaq individual (the Paleo-Eskimo era in Greenland) was relatively not young and, probably, about 35–40 years at death [5].

## CONTAMINATION CHALLENGE OF ANCIENT DNA

Contamination of material with bacterial DNA is a significant obstacle when working with ancient DNA. It has recently been shown that the epigenetic characteristics of vertebrates (methylation of CpG) can be used for the separation of bacterial and ancient human genomic DNAs [17]. Methylated CpG-dinucleotides are found only in somatic vertebrate cells. Bacterial genome also contains methylated cytosines and adenines, but not in CpG. The MBD protein family contains a methyl-DNA-binding domain (MBD), which binds methylated DNA containing single methylated CpG [18]. Affinity chromatography using MBD domains is a method routinely used for constructing the methylation maps of the genomes of different organisms. This method allows one to both unravel ancient methylomes and separate the DNA of ancient vertebrates and microorganisms. With the example of the Paleo-Eskimo Saqqaq individual, woolly mammoths (Yuka and Khroma), polar bears, and two equine species it was shown that DNA methylation survives in a variety of tissues, environmental contexts, and over a long temporal range of remains emergence (4,000 to 45,000 years BC). MBD enrichment affinity chromatography allows one to analyze ancient microbiota, as well as potentially pathogenic genomes [17].

## TRANSCRIPTIONAL ACTIVITY OF ANCIENT GENES AS INFERRED FROM NUCLEOSOME MAPS

**Fig. 2:**
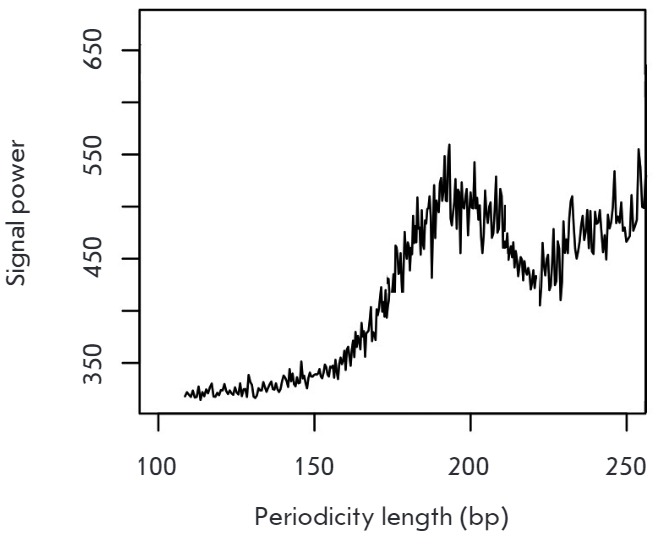
Graph of the spectral density (power spectral estimation). Analysis of the region around genes transcription starts (+/- 1000 bp), correction with respect to the distribution of background readings in the analysis of the DNA of modern elephants was performed. We used the genome sequencing data of a baby mammoth found in 2009 in the downstream of the Khroma River (Prokhorchuk E.B. et al., unpublished data). The age of the finding exceeds 50,000 years.

DNA methylation can serve as a marker of gene transcription repression, but the information is insufficient in order to establish whether the gene was expressed or not. To predict the transcriptional activity of a gene, more information is needed, such as histone modification, chromatin structure, and the transcription factors binding to the regulatory regions. The first attempts to work with ancient proteins have been made [19]. The sequencing of ancient DNA revealed an unexpected source of epigenetic information. L. Orlando *et al*. found periodicity in the nucleotide read depth [5]. The authors hypothesized that, instead of being the result of sequence alignment errors or sequencing artifacts, the observed periodicity patterns of the nucleotide read depth could be associated with the protection of DNA by nucleosome binding with a degradation of the linker regions either by DNases that enter the nucleus during cell death or by post-mortem strand breaks. In such a scenario, the observed read depth reflects nucleosome occupancy. Spectral methods of DNA analysis are used to search for hidden periodicities. Thus, in the case of relatively short sequences a Fourier transformation allows one to derive statistical criteria with a self-averaging property [20]. The application of the Fourier transform to the function that correlates the read depth with a coordinate in the genome reveals a strong peak at 180–190 nucleotides, indicating that the periodicity of coverage in sequencing coincides with the periodicity of chromatin organized into nucleosomes (*Fig. 2*). When analyzing the distribution of the 5’ read depth, it turned out that the distances of a characteristic length of 100 nucleotides were associated with the periodicity of 10 nucleotides, coinciding with the length of a turn of the DNA helix: the nucleotides facing the nucleosomes will not mark the start of readings, because they are less available to nucleases. In ancient DNA, the positioning of nucleosomes stretching over 4 kbp around the CTCF binding sites was mapped with high precision and the nucleosome location negatively correlated with the DNA methylation levels. Actively transcribed areas can be found by an analysis of the DNase I hypersensitive sites located at the start of transcription [21]. It is assumed that open chromatin state regions are more sensitive to DNase I cleavage during apoptosis or after the death of an organism. Therefore, during full genome sequencing the read depth at the start of active gene transcription will be reduced in comparison with silent genes. Simultaneous consideration of the read depth in the region of a certain transcription start and information on DNase I hypersensitive sites from the ENCODE database opens up a possibility for determining the transcriptional activity of the respective genes.

## PROSPECTS

By combining information on DNA methylation and DNase I hypersensitivity sites at transcription start sites we might be able to reconstruct the quantitative expression pattern of genes in ancient samples in the near future. If we are lucky to find a well-preserved fossils of the human brain, that will be a breakthrough in the study of ancient DNA and human evolution. Such a prospect is supported by the recent discovery of a woolly mammoth with well-reserved brain structures [22]. It is assumed that the main differences in the higher nervous activity of ancient and present-day humans will be located at the epigenetic level [23, 24].

## Abbreviations

MpG: methylated CpG
PMD: partially methylated domain
DMV: DNA methylation valley
MBD: methyl DNA binding domain
